# Elderly age and male gender as risk factors for Non Diagnostic cytology at thyroid fine needle aspiration: results of a large cytological series

**DOI:** 10.1007/s12020-026-04565-z

**Published:** 2026-03-09

**Authors:** Laura Croce, Spyridon Chytiris, Marsida Teliti, Isabella Chiardi, Francesca Coperchini, Linda Loretta Businaro, Flavia Magri, Tshering Dorji, Mario Rotondi

**Affiliations:** 1 Department of Internal Medicine and Therapeutics, University of Pavia, Pavia, 27100 Italy; 2 Unit of Endocrinology and Metabolism, Laboratory for Endocrine Disruptors, Istituti Clinici Scientifici Maugeri IRCCS, Pavia, 27100 Italy; 3 Unit of Anatomic Pathology, Istituti Clinici Scientifici Maugeri IRCCS, Pavia, 27100 Italy

**Keywords:** Non diagnostic, Thyroid cytology, Fine needle aspiration, Gender, Age

## Abstract

**Purpose:**

Non-diagnostic (ND) result of ultrasound-guided fine-needle aspiration cytology (FNAC) of thyroid nodules can lead to diagnostic delays, repeated procedures and, potentially, unnecessary surgeries. This study aimed to evaluate the role of patient age and gender as predictors of a ND cytological result of FNAC, both at first sampling and after repeat procedures.

**Methods:**

We retrospectively analyzed 5,774 FNACs performed between October 2017 and April 2025 at a single tertiary center. All procedures were conducted by an experienced endocrinologist and interpreted by a single expert cytopathologist. ND rates were compared by age and gender. Logistic regression assessed independent associations, and interaction analysis evaluated whether age effects differed by gender.

**Results:**

ND cytology occurred in 29.5% of cases. Patients with ND results were older (59.3 ± 14.2 vs. 57.3 ± 14.0 years, *p* < 0.001) and more often male (29.6% vs. 23.0%, *p* < 0.001). Logistic regression confirmed male gender (OR 1.376, 95% CI 1.211–1.563) and age (OR 1.009, 95% CI 1.005–1.013) as independent predictors (*p* < 0.001 for both), with a significant age × gender interaction (*p* = 0.036), showing a stronger age effect in males. Among 443 patients repeating FNAC after an initial ND result, 39% remained ND, with no significant age or gender differences between persistent-ND and diagnostic outcomes.

**Conclusion:**

Elderly age and male gender independently increase the likelihood of ND FNAC results. These factors should be considered when planning thyroid FNAC, although they do not predict persistent ND outcomes upon repeat sampling.

## Introduction

Non-Diagnostic (ND) or “unsatisfactory” thyroid cytology specimens (referred to as Bethesda I in the Bethesda classification or TIR 1 in the Italian Cytopathology Classification) include specimens with poor cell preservation, blood contamination, or an insufficient number of follicular cells [1, 2]. The rate of ND results for Ultrasound-guided Fine-Needle Aspiration Cytology (FNAC) varies between studies, ranging from 8% up to 46% [3–5]. Given the non-negligible risk of malignancy in thyroid nodules with a ND FNAC submitted to thyroidectomy [3] all the latest guidelines (including the American Thyroid Association guidelines [6], European Thyroid Association (ETA) guidelines [7], Korean Thyroid Association guidelines [8] and British Thyroid Association guidelines [9] suggest to repeat FNA in nodules with a non-diagnostic result.

ND result of FNAC can have a relevant impact on sanitary costs and patient’s well being, since they can lead to a delay in thyroid cancer diagnosis, repeated procedures (sometimes multiple times) and, potentially, unnecessary surgeries. It is thus relevant to identify possible predictive factors for a ND result in order to develop preventive strategies and to provide correct information to patients.

Previously established factors driving a higher risk for ND cytology, include: (i) those related to specific US characteristics of the thyroid nodule, such as size, vascularization, echogenicity, presence of calcifications and cystic nature [10–13]; (ii) those related to the operator expertise and technical issues, such as smear preparation technique, use of core needle biopsy or in-situ cytological evaluation bedside or rapid onsite sample evaluation [4, 10, 14, 15].

While the role of nodule characteristics and operator expertise appear fully elucidated and rather expected factors, the role of gender and age appears less straightforward.

Indeed, discrepancy exists as to the impact of age and gender on the rate of ND results, with one study suggesting that male gender and elderly age may increase the risk of ND results [13], and other studies not confirming such an association [14]. Thus, further evaluation of the association between gender and age with the rates of ND cytology may be of not negligible interest.

The aim of the present study was to assess the role of patient’s age and gender on the rate of ND result in a large cohort of patients undergoing FNA both at the first FNAC and after repetition of the sample.

## Materials and methods

All patients undergoing thyroid cytology sampling by Fine Needle aspiration in the Unit of Endocrinology of ICS Maugeri and analyzed by the Unit of Pathology of ICS Maugeri from October 2017 to April 2025 were retrieved.

**Fig. 1:**
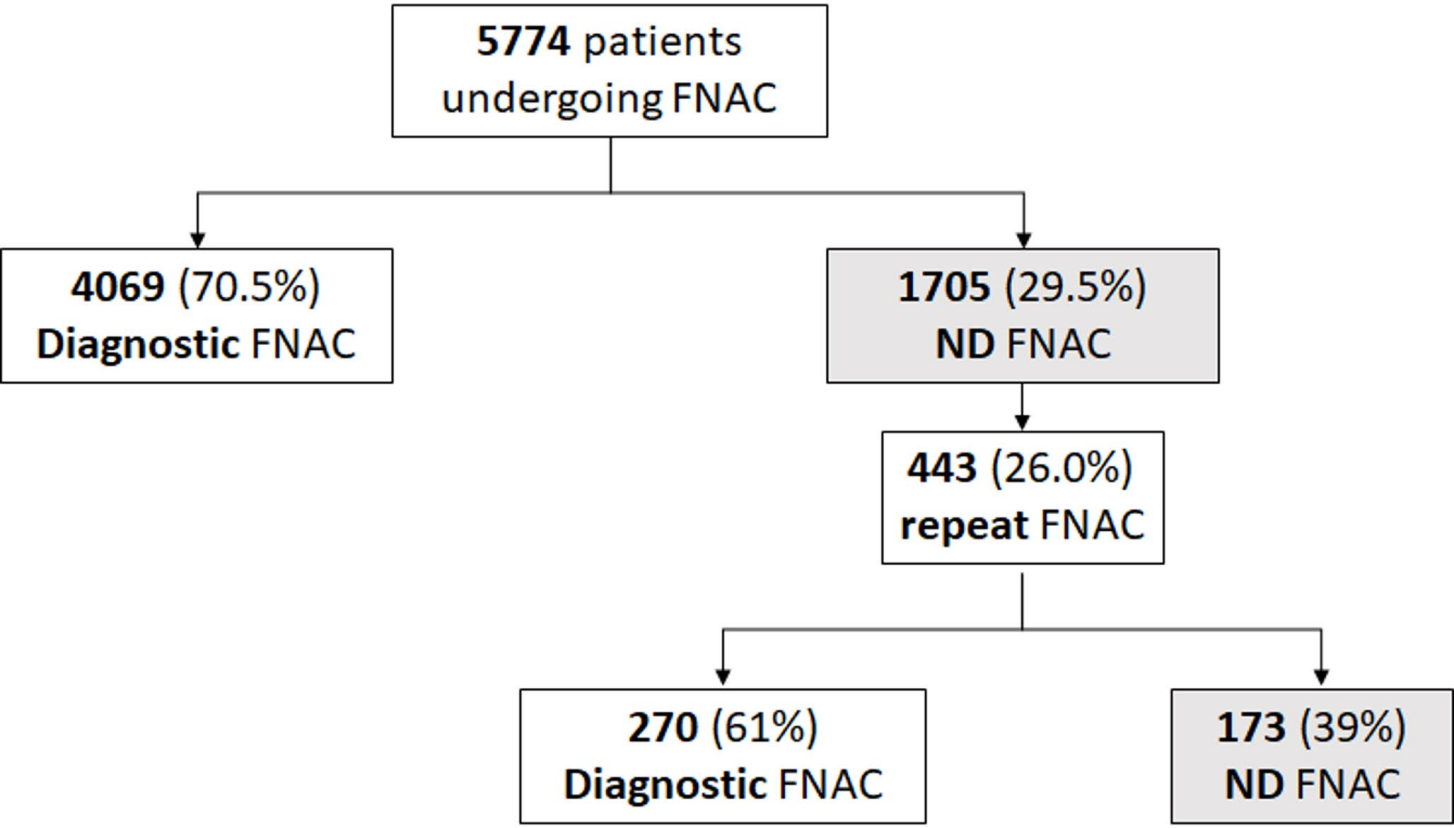
flow-chart of the study. FNAC: Fine Needle Aspiration Cytology, ND: Non diagnostic

The study group included 5774 patients (4336 females, 75.1%, and 1438 males, 24.9%) with a mean age of 57.9 ± 14.1 years. As shown in Fig. 1, patients were stratified in two groups according to a ND cytological results (Group I *n* = 1705) and patients with any other cytological result (Group II *n* = 4069). FNAC was repeated in 443 patients (26%) from Group I.

In order to minimize potential bias due to the operator expertise: (i) thyroid FNABs were performed under US guidance by an expert endocrinologist with more than 20 years’ experience in thyroid ultrasound and FNA; (ii) on slides stained with May-Grunwald Giemsa and monolayer ThinPrep slides, cytology was interpreted by one experienced cytopathologist using the ICCRTC criteria [2]. A 23 Gauge Needle with an aspiration technique was employed. Two passes were performed for each nodule.

The decision to repeat FNAB after a first ND result was made by the clinician, based on clinical and US characteristics. During the study enrollment time, the adherence to the EU-TIRADS criteria gradually increased over time, moving from a size-threshold approach (approximately 10 mm) to an US-characteristics guided approach.

Data regarding age and gender were collected for all patients.

### Statistical analysis

Statistical analysis was performed using the SPSS software (SPSS, Inc., Evanston, IL). Continuous variables were expressed as mean ± standard deviation or as median and range when appropriate and comparison between groups was performed using the Student’s t test for unpaired data and the Mann–Whitney U-test according to a normal or a non-parametric distribution. Qualitative data were expressed as frequencies. Frequencies among groups were compared using the χ2-test with Fisher’s correction when appropriate. A logistic regression model was designed including the rate of ND results as dependent variable and age, gender as covariates. To examine whether the effect of age on the likelihood of ND was moderated by gender, a logistic regression model including age, gender and their interaction term (age × gender) was fitted. A *p* value < 0.05 was considered statistically significant.

## Results

As shown in Table 1, patients from Group I were characterized by older age (59.3 ± 14.2 in Group I vs. 57.3 ± 14.0 in Group II, *p* < 0.001) and higher male gender prevalence (29.6% in Group I vs. 23.0% in Group II, *p* < 0.001).

**Table 1:** Comparison of patients with a Non-diagnostic vs. Diagnostic FNA result

	Non-diagnostic	Diagnostic	*p*-value
N	1705 (29.5%)	4069 (70.5%)	
Age	59.3 ± 14.2	57.3 ± 14.0	< 0.001
N of males (%)	504 (29.6%)	934 (23.0%)	< 0.001

In order to evaluate the relationship between age and rates of ND result, the rates of ND versus other cytological classes were compared according to age quartiles. A progressive and significant increase in the rate of ND result was observed from the lowest to the highest quartile (*p* < 0.001 for trend).

**Fig. 2:**
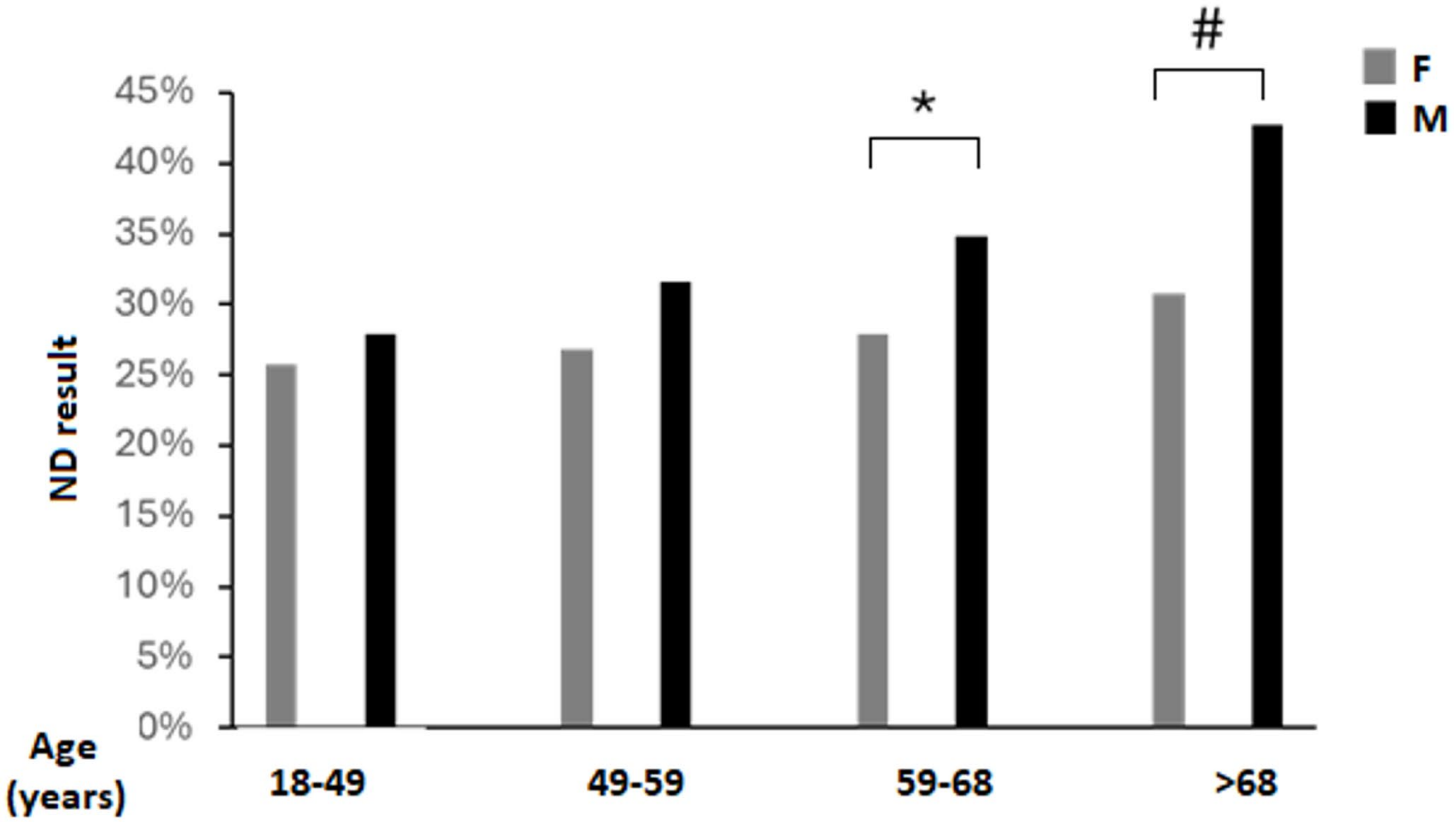
rate of Non Diagnostic (ND) result in Males and Females according to age quartiles. * *p* < 0.05, # *p* < 0.001

To further confirm this finding a comparison of gender ratio according to age quartiles was performed. As shown in Fig. 2, while no differences in the rates of ND could be observed between Males and Females in the first two quartiles of age, the difference was significant in the two highest quartiles.

**Fig. 3:**
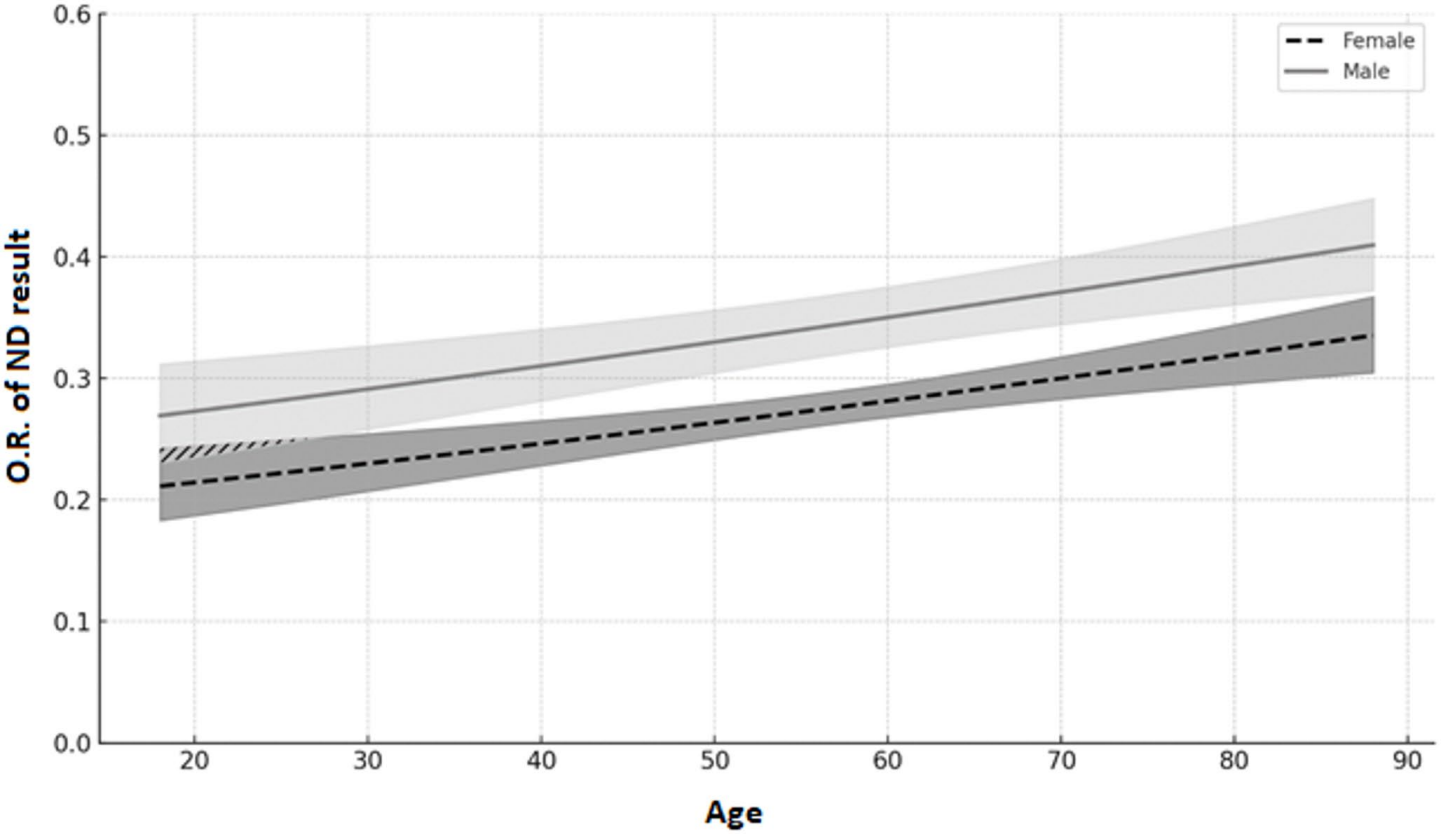
Logistic regression model predicting the probability of a ND cytological outcome based on patient age and gender. The dashed line represents predicted Odds Ratio (O.R.) for females, and the solid line for males. Shaded areas indicate 95% confidence intervals. The Y-axis is truncated at 0.6 for better visualization of the differences between groups

Logistic regression analysis showed that male gender [O.R. 1.368 (C.I. for O.R. 1.203–1.555); *p* < 0.001] and age [O.R. 1.009 (C.I. for O.R. 1.005–1.013); *p* < 0.001] were both significant and independent factors associated with a non-diagnostic cytological result (Fig. 3).

To assess whether there was any interaction between gender and age, a moderation analysis was performed, designing a regression model that included age, gender and their interaction as dependent variables and ND result as dependent variable. The results showed a significant interaction between gender and age in influencing the rate of ND results (O.R. for interaction 1.010 (CI 1.001–1.020), *p* = 0.036), suggesting that the effect of age was different according to patient’s gender. In particular, the effect of age, although significant in both genders, was stronger in males [O.R. 1.017 (CI 1.008–1.025), *p* < 0.001] than in females [O.R. 1.006 (CI 1.002–1.011), *p* = 0.009]

As shown in Fig. 1, among Group I patients who had an ND result at the first sampling, 443 patients (26%) repeated the FNAC. No differences were noted between patients repeating or not-repeating FNAC in terms of percentage of males (28.2% vs. 30.0%, *p* = 0.471) nor age (58.5 ± 14.0 vs. 59.5 ± 14.3 years, *p* = 0.191). In 173 patients (39%) out of 443 the FNAC repetition still showed a ND result. On the other hand, in 270 patients out of 443 (61%) the repetition led to a non-ND result.

In details, 146 (54.1%) reached a Benign result (TIR2), 86 (31.9%) reached a low-risk indeterminate (TIR3A) result, 22 (8.1%) reached a high-risk indeterminate (TIR3B) result, 3 (1.1%) reached a suspicious (TIR4) result and 8 (3%) reached a malignant (TIR5) result. Of note, no significant differences in terms of age (60.0 ± 14.1 vs. 59.2 ± 14.0, *p* = 0.547) and rates of male gender (31.8% vs. 25.9%; *p* = 0.181) were observed between the 173 patients with a persistent ND result as compared to the 270 with a non-ND result.

## Discussion

The present study was specifically designed to evaluate the role of age and gender as potential predictors of a ND results in patients undergoing FNAC for thyroid nodules. The results indicate that both age, particularly over 68 years-old, and male gender, in a significant and independent manner, are associated with a higher rate of ND results. The issue of the potential relationship between cytological outcome and age and gender was addressed in a few previous studies, reporting contrasting findings.

In detail, one previous study evaluated the impact of age in raising the frequency of ND result reporting a positive association [16]. Studies evaluating both age and gender yielded different results. Indeed, most studies did not find any significant relationship between age or gender and the rates of ND [11, 14, 17–19]; one study reported a correlation between patient’s age, but not gender, and rate of nondiagnostic cytology [20] and one other [13] reported a significant role for both age and male gender.

These results would appear in agreement with those obtained by Grani et al. [13]. However, overviewing some differences between the present and previous studies addressing this topic might be helpful for explaining the discrepancy:

i) The mean age of included patients in the previous studies appears to be rather lower than in the present study, which has likely reduced the effect of age in the previously studied cohorts. Just to give an example, Alexander et al. evaluated a series of patients with nearly 10 years lower mean age as compared to the here present cohort [14].
ii) Sample size might also have played a role. The present study and the one by Grani et al. [13], who reported an association with both age and gender, were both conducted on large series.

The sample size of the present study, which is the largest so far, allows for more nuanced comparisons on this topic. Moreover, previous studies did not evaluate the interaction of gender and age and did not assess the role of these factors on the repetition of the exam.

The results of the present study show that while age and gender are predictive of the first ND result, these factors were not significantly related with a higher risk of a persistently ND result after repetition of the exam. These data would support the role of procedure-related factors (such as pain or discomfort during the procedure, greater thickness of neck muscles, or problems in neck extension) in determining the higher risk of ND results in male and elderly subjects. These procedural problems can be in part overcome by modifying FNAC technique (for example through multiple and/or more extensive sampling). These results would support the indication to modify the sampling approach in “high risk patients”, such as elderly male patients, with more passes and use of rapid on-site evaluation. These findings are in apparent contrast with the results of a recent study by Cosme et al. on a group of nodules with a first ND result in which the FNA was repeated, showing that male gender and age were predictive of a persistently ND result [21]. In this study, the rate of a persistently ND result was similar to our series (32%). Besides age and gender, the use of antiplatelet/anticoagulant drugs, was found to be associated with a repeated ND result.

The rate of ND cytological results in our series is rather high (29%) and higher than what is considered acceptable by international guidelines (20% by American Thyroid Association guidelines [6], 15% by British Thyroid Association Guidelines [9], 11% by European Thyroid Association guidelines [7]).

Given the retrospective nature of this study, we can provide some tentative explanation for this high rate of ND.

- A significant percentage of our patients present with mixed/cystic nodules typical of multinodular goiter due to past iodine deficiency [22].
- Rapid on-site cytology review is not systemically employed in our center.
- While most scientific societies recommend to keep the rate of ND results below 20%, it should be acknowledged that in the previously described largest patient series higher rate of ND were observed (~ 36%) [13].
- The mean age of the patients included of the largest previous studies (55.8 years for Grani et al. [13]and 48.2 years for Alexander et al. [14]) is lower than our series (57.9 years). The older age in our series may have contributed to a higher rate of “difficult patients”, due to limited neck extension, increased frequency of fibrotic or calcified nodules, and a greater prevalence of antiplatelet or anticoagulant therapies, all of which may hamper adequate sampling.
- Our center is a referral one for several territorial-based centers. Indeed, more than 40% of patients undergoing FNAC were referred from external centers. Given that the results of FNAC can vary significantly between low- and high-volume centers [15], it is likely that a higher rate of “difficult” cases was referred to our center.

Nevertheless, it should be noted that the high rate of ND cytology would not seem to have played a role in influencing the results. Indeed, the results of the logistic regression analysis confirmed that the two variables taken into account in the present study (i.e. age and gender) were found to be independently and in a significant manner related to a ND cytology. The core results of this study can thus be considered valuable also in a clinical setting with a lower rate of ND.

In our center, a rather low repetition rate for ND results was observed (26%). Also in this case, due to the retrospective nature of the study, we cannot provide a full explanation for this finding. Nevertheless, this can be in part explained by the presence of partially or fully cystic nodules, in which the repetition rate is significantly lower. Moreover, since our center is a referral one, a large percentage of patients were referred from other Endocrine Units on the territory. In these latter patients, the rate of repetition is usually lower. This could be due to either the fact that following the ND result the exam was repeated in another center, or to the possibility that the patients were addressed to our Unit following a previous ND result. Lastly, since the beginning of the study EU TI-RADS classification was increasingly adopted in our Center [23]. As a result, some of the early enrolled patients may have not repeated FNAC as they did not meet the EU TI-RADS criteria (which was more precisely employed throughout the years). Nevertheless, the fact that no significant differences in terms of age and gender could be observed between those who repeated vs. those who did not repeat FNAC supports the lack of any major selection bias.

Due to its retrospective nature and to the fact that almost half of patients were referred from external centers, we could not collect relevant information regarding several clinical and anthropometrical factors that may explain the observed effect of male gender and older age on ND results rate.

To give a few examples:

- Men and elderly subjects receive more frequently antiplatelet/anticoagulant therapies [24, 25]. Indeed, male patients with thyroid nodules tend to have a higher rate of cardiovascular and metabolic comorbidities [26, 27]. The role of this kind of therapies in determining a higher rate of ND was assessed by previous studies, with contrasting results. While some studies showed a higher rate of ND in patients taking aspirin [21, 28], another study failed to show this association [29].
- No study specifically assessed the role of BMI or obesity in determining a higher risk of ND results. Nevertheless, since, at least in Italy, BMI tends to be higher among males [30], this could possibly be a factor involved in the higher risk of ND among male subjects.
- Similarly, neck thickness was not evaluated by any study as a risk factor for ND results. Nevertheless, it is largely demonstrated that men have a higher neck thickness, as assessed by higher circumpherence and greater coronal/sagittal width. Moreover, men tend to have greater fat deposits in the neck area compared to women [31, 32]. This factor could be and intriguing mechanism explaining the association between ND and male sex.

Future prospective studies specifically addressing the above reported potential confounders are required.

As a further potential limitation, the present study is single-center one, potentially limiting the generalization of the here-reported results. Nevertheless, if from one side this could represent a limitation, from the other the fact that the same operator has performed all FNAC procedures might be regarded as a benefit, in that it has reduced operator and procedure-dependent variability.

In conclusion, the results of the present study demonstrate that male gender and age significantly and independently increase the risk of a ND result in FNA. This information should be taken into account when performing FNA in elderly subjects, especially if males, in order to reduce the risk of a non-diagnostic result. On the other hand, age and gender are not predictive of repeated non-diagnostic results, and repetition of the exam leads to a diagnostic result in most cases.

## Data Availability

The data that support the findings of this study are not openly available due to reasons of sensitivity and are available from the corresponding author upon reasonable request.
